# Development of Stable Chimeric IL-15 for Trans-Presentation by the Antigen Presenting Cells

**DOI:** 10.3389/fimmu.2021.646159

**Published:** 2021-04-19

**Authors:** Manoj Patidar, Naveen Yadav, Sarat K. Dalai

**Affiliations:** ^1^Institute of Science, Nirma University, Ahmedabad, India; ^2^Department of Zoology, Govt. College Manawar, Dhar, India; ^3^Translation Health Science and Technology Institute, NCR-Biotech Science Cluster, Faridabad, India

**Keywords:** IL-15, bioavailability, immunotherapy, adjuvant, memory T cell

## Abstract

IL-15 is one of the important biologics considered for vaccine adjuvant and treatment of cancer. However, a short half-life and poor bioavailability limit its therapeutic potential. Herein, we have structured IL-15 into a chimeric protein to improve its half-life enabling greater bioavailability for longer periods. We have covalently linked IL-15 with IgG2 base to make the IL-15 a stable chimeric protein, which also increased its serum half-life by 40 fold. The dimeric structure of this kind of IgG based biologics has greater stability, resistance to proteolytic cleavage, and less frequent dosing schedule with minimum dosage for achieving the desired response compared to that of their monomeric forms. The structured chimeric IL-15 naturally forms a dimer, and retains its affinity for binding to its receptor, IL-15Rβ. Moreover, with the focused action of the structured chimeric IL-15, antigen-presenting cells (APC) would transpresent chimeric IL-15 along with antigen to the T cell, that will help the generation of quantitatively and qualitatively better antigen-specific memory T cells. *In vitro* and *in vivo* studies demonstrate the biological activity of chimeric IL-15 with respect to its ability to induce IL-15 signaling and modulating CD8^+^ T cell response in favor of memory generation. Thus, a longer half-life, dimeric nature, and anticipated focused transpresentation by APCs to the T cells will make chimeric IL-15 a super-agonist for memory CD8^+^ T cell responses.

## Introduction

Antigen-specific CD8^+^ T cells generated either by the natural infection or through vaccination play a critical role in the clearance of intracellular pathogens such as TB, Malaria, HIV, HCV, and influenza (1). In the treatment of cancers, the anti-tumor activity of CD8^+^ T cell, and CAR (chimeric antigen receptor) T cell therapy has opened new avenues. Generation of CD8^+^ T stem cell memory (T_SCM_) is reportedly known as an ideal source for the maintenance of long-lasting antitumor T-cell responses (2). IL-15 has been shown to improve the antitumor potency of T cells as well as the generation and self-renewal of T_SCM_ (2). While inducing robust T cell response by vaccines is somewhat achievable, the generation of memory T cells and their long-lasting maintenance, and reactivation at the time of infectious challenge or re-infection is very challenging. The literature documents the role of IL-15 in overcoming the above-mentioned challenges (3). IL-15 as vaccine adjuvant enhances the protective immunity against infectious pathogens, including influenza virus, *Toxoplasma gondii*, *Herpes simplex virus*, *Trypanosoma cruzi*, and complex intracellular protozoan pathogen by boosting the survival and improving cytotoxic activity of T and NK cells as well as IFN-γ production (4–9). Co-administration of plasmid expressing IL-15 along with HIV-DNA vaccine has shown to generate the higher frequency of CD8^+^ Tem cells (10). The improved efficacy of the HIV gp160 vaccine (i.e., *Vaccinia* expressing HIV gp160) by augmenting the generation and maintenance of long-lasting CD8^+^ T cells has been reported (11). Among the twelve anti-cancer immunotherapy drugs, IL-15 was ranked first by the National Cancer Institute, NIH, USA in 1997 (12, 13). Therefore, many clinical trials are being conducted to establish IL-15 as a vaccine adjuvant and an important biologics for anti-cancer immunotherapy (3). However, exploiting its maximum therapeutic potential is still a challenge due to its shorter half-life, poor bioavailability, and unstable nature.

Several approaches have been adopted to improve the stability and half-life of immune modulators including cytokines. IL-15 is known to bind naturally with its receptor IL-15Rα and presented in cis-or trans-manner to the cells bearing receptor IL-15Rβ/γ_c_ (common γ chain) (3) prompting investigators to use IL-15Rα as a base for stabilizing IL-15 moiety. Mortier et al. linked IL-15 with IL-15Rα or with sushi domain (IL-15 binding domain of IL-15Rα, sIL-15Rα) and made soluble IL-15-IL-15Rα complexes with an objective to bind it to cells expressing IL-15Rβ/γ_c,_ and thereby promoting cell proliferation and anti-apoptotic activities (14). In an alternative strategy, IL-15 was non-covalently linked with IL-15Rα-IgG1 fusion proteins to make the overall complex more stable (15, 16). The severe toxicity has also been reported in mice treated with the modified IL-15 because of the hyper-activation of NK cells (17).

We have structured a chimeric protein to achieve the desired efficacy of IL-15, with minimal adverse effects. In addition to increasing the half-life, our study aimed at improving the efficacy of IL-15 with respect to augmenting Ag-specific memory T cell response by exclusively directing IL-15 to T cells through APCs. As transpresentation is the dominating mode for the action of IL-15 and is required for the reactivation of memory CD8^+^ T cells during the infectious challenge (18), we strategically designed/structured a stable and long-lasting IL-15 that could be predominantly presented by professional APCs to T cells. Therefore, IL-15 was systematically transformed into T cell centric adjuvant by covalently linking with IgG2/2a heavy constant chain (IgHg2/2a) that would form a stable complex, and be targeted specifically to APC and thus presenting IL-15 to T-cells with MHC/p complex. Both molecules were fused through a linker to avoid any type of steric hindrance. Additionally, in the chimeric IL-15, we have introduced N72D mutation in the distal region of human IL-15 to improve its binding affinity with the IL-15Rβ present on T cells. And to delay the immune clearance, we have introduced K322A mutation (site for complement binding) in the immunoglobulin-heavy constant chain to increase the residence time of chimeric IL-15 in the periphery. Thus, the structured chimeric IL-15 when expressed in CHO cells naturally forms the dimer of IL-15-IgG2/2a. It is very stable with Tm >70.00°C and, its serum half-life is increased by >40 folds. Our data also suggest that the engineered chimeric IL-15 is biologically active and will generate robust antigen-specific T cell responses.

## Results

### Engineered Chimeric IL-15 Favors Dimerization: *In Silico* Evaluation

**Figure 1:**
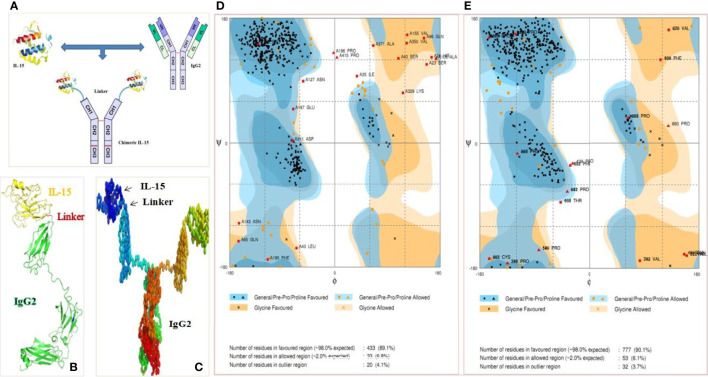
Ig based chimeric IL-15 design. **(A)** Anticipated structure of chimeric IL-15 upon covalently linking IL-15 with IgHG2/2a. **(B, C)** Amino acid (AA) sequences of human or mouse IL-15 in combination with AA of huIgHG2 or mIgHG2a (NtoC) was submitted to I-TASSER **(B)** and COTH server **(C)** for prediction of monomeric and dimeric structures respectively. **(D, E)** Ramachandran map of monomeric chimeric IL-15 **(D)** and dimeric chimeric IL-15 **(E)** generated by submitting the structures to Rampage server.

The superiority of homodimeric Ig based biologics over their monomeric counterparts has been well-established (19–21). However, achieving dimerization of chimeric molecules is very challenging. Therefore, we performed first the systematic *in silico* evaluation to fulfill the prerequisite of homodimerization of chimeric IL-15 (22). Different combinations of various domains of Ig and sequences of linkers with IL-15 were fused and submitted for the prediction of homodimer formation. Having tried out various combinations, we found the IL-15-GS-linker-IgHg2/2a (**Figures 1A, B**) not only fulfils the requirements for homodimer formation, but also helps to maintain its native structure keeping the IL-15 molecules apart and available for interaction (**Figure 1C**). Then we assessed various properties of homodimeric chimera and compared them with their monomeric forms to attest to the formed structure. The favorable and allowed residues in the Ramachandran map were calculated 89.1 & 6.8% for monomer, and 90.1 & 6.1%, respectively for homodimer structures (**Figures 1D, E**). The data suggest that the engineered structures are favorable. The instability indices were estimated <40 confirming the stability of the formed structure. In addition, a clash score of 0.00 is an indication of the fact that there was no unfavourable overlap of electron clouds. This further confirms that the packaging of the proteins is tight. Cβ deviations (0.00) and MolProbity score (95–99 percentile) suggests the stability and usefulness of constructed chimeric IL-15.

### Chimeric IL-15 Has a Higher Binding Affinity Towards IL-15 Rβ

Next, we determined the ability of chimeric IL-15 to interact with its receptor β (IL-15Rβ) through protein-protein docking. The number of hydrogen bonds and salt bridges were seen higher in the chimeric IL-15-receptor docking compared to the native IL-15-receptor docking. These interactions also suggest that the IL-15 portion of the chimera is able to interact with its receptor β efficiently. Moreover, we found that IgG2/2a, a portion of chimera, does not interact with the IL-15 receptor, and the IL-15 portion of chimera binds exclusively to its own receptor β (22).

One of our aims to make this chimeric IL-15 is to facilitate the efficient binding of IL-15 moiety to IL-15 Rβ. To test whether the designed chimera improves the binding affinity of IL-15 to Rβ, we predicted and compared this with native IL-15 and IL-15 with N72D mutation. We estimated the binding free energy (Gibbs free energy, ΔG) as docking algorithm provides the docking score of protein–protein complexes based upon ΔG. The lesser binding energy is confirmatory of the stability of the protein-protein complex. The ΔG of native IL-15 and N72D IL-15 with the Rβ was calculated as −8.7 kcal mol^−1^ and −9.6 kcal mol^-1^, respectively. On the other hand, ΔG of chimeric IL-15 with the same Rβ was -73.2 kcal mol^−1^ (**Table 1**). Based on these results, the chimeric IL-15–Rβ complex is considered as a stable complex compared to the native IL-15–Rβ complex.

**Table 1:** Prediction of the binding affinity.

Sr. No.	Docked Structure (Protein-protein complex)	Protein-Receptor	ΔG (kcal mol^−1^)	K_d_ (M) at 25.0°C
1	IL-15-IL-15 Tri-receptor complex	IL-15-IL-15 Rα	−9.7	7.7E−08
2	IL-15-IL-15 Tri-receptor complex	IL-15-IL-15 Rβ	−8.7	4.5E−07
3	IL-15-IL-15 Tri-receptor complex	IL-15-IL-15 Rγ	−10.7	1.5E−08
4	N72D IL-15-IL-15 Tri-receptor complex	IL-15-IL-15 Rβ	−9.6	9.0E−08
5	N72D IL-15-IL-15 Tri-receptor complex	IL-15-IL-15 Rγ	−10.6	1.7E−08
6	Chimeric IL-15 (wild)-Receptor βγ complex	IL-15-IL-15 Rβ	−73.2	1.9E−54
7	Chimeric IL-15 (wild)-Receptor βγ complex	IL-15-IL-15 Rγ	−8.6	4.8E−07
8	Chimeric IL-15 (N72D)-Receptor βγ complex	IL-15-IL-15 Rβ	−73.7	8.7E−55
9	Chimeric IL-15 (N72D)-Receptor βγ complex	IL-15-IL-15 Rγ	−8.6	5.0E−07
10	Chimeric IL-15 (N72D_K322A)-Receptor βγ complex	IL-15-IL-15 Rβ	−73.7	8.9E−55
11	Chimeric IL-15 (N72D_K322A)-Receptor βγ complex	IL-15-IL-15 Rγ	−8.6	5.1E−07

Prediction of the binding free energy (Gibbs free energy) ΔG and the dissociation constant Kd for various complexes suggest that chimeric IL-15 has higher binding affinity towards IL-15Rβ.

Similarly, prediction of dissociation constant (Kd) also suggests that chimeric IL-15 would bind to receptor β with higher affinity (Kd = 8.9E−55) compared to that seen with native IL-15 (Kd = 4.5E−07) and N72D IL-15 (Kd = 9.0E−08) (**Table 1**).

### Cloning of IL-15 and IgG2 Constant Heavy Chain (IgHg2) Genes

**Figure 2:**
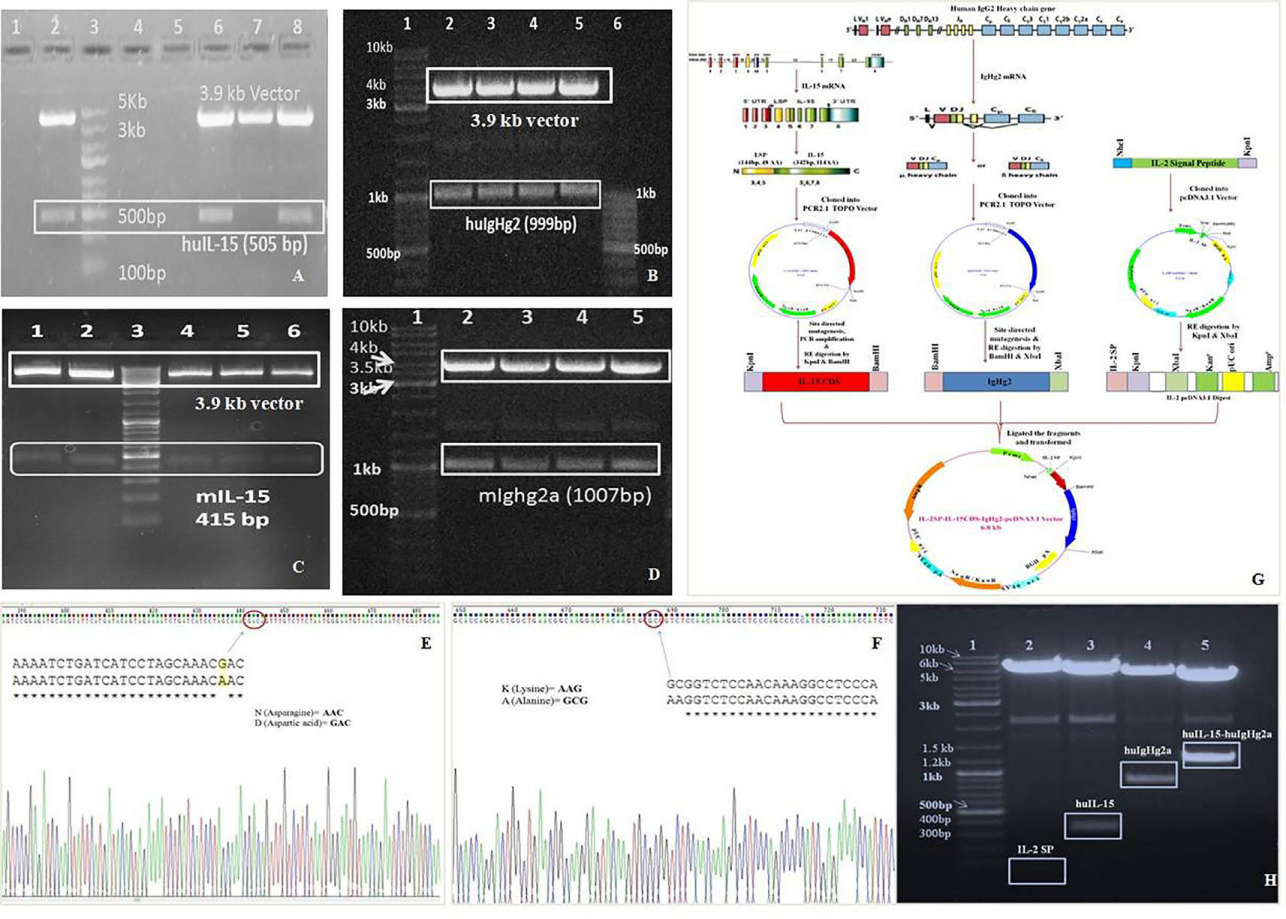
Development of chimeric IL-15. **(A–D)** In wet lab experiments, IL-15 and IgHG2/2a genes from mouse and human were separately cloned into PCR2.1 TOPO vector. The EcoRI digestion of respective vector showed two bands i.e., empty vector (upper band) as well as the inserts: insert huIL-15 **(A)**, huIgHG2 **(B)**, mIL-15 **(C)** and mIgHG2a **(D)**. **(E, F)** N72D mutation in huIL-15 portion **(E)** and K322A mutation in huIgG2 Fc **(F)** introduced by site directed mutagenesis were confirmed by Sanger sequencing. **(G)** The physical map of Fusion strategy utilized in this study. **(H)** Sequentially huIL-15 and huIgHG2 genes were inserted into IL-2-pcDNA3.1 vector; restriction digestion analysis for chimeric IL-15 construct was carried out with various combinations of restriction enzymes to confirm the inserts.

huIL-15 (505 bp; CDS with signal peptide; without stop codon), mIL-15 (483 bp) (without stop codon), huIgHg2 (981 bp; CH1-Hinge-CH2-CH3; with stop codon), and mIgHg2a constant heavy portion (1,007 bp; with stop codon) were separately cloned into PCR2.1 TOPO vector. Resultant plasmids were digested by EcoRI enzyme to confirm the respective inserts. For example, two bands were obtained i.e., upper band at 3.9 kb and lower band at 505 bp representing TOPO vector and IL-15 insert, respectively with IL-15-TOPO vector, (**Figure 2A**). Further confirmation of the inserts was carried out by successful amplification of 505 bp insert by PCR and sequencing. FASTA sequences obtained from Sanger sequencing of inserts IL-15 and IgHg2 were compared and found 100% similar to the original sequences by the NCBI blast and SIM alignment. Similarly, mIL-15-TOPO, huIgG2-TOPO, and mIgG2a-TOPO cloning were confirmed where we obtained a 415, 999, and 1,007 bp insert respectively for mIL-15, huIgHg2, and mIgHg2a (**Figures 2B–D**).

### Site Directed Mutagenesis and Fusion Gene Construction

While IgG2/2a base was used for the presentation of IL-15 by APCs to T cells, we wanted to improve the affinity of IL-15 with its receptor β enabling optimum T cells signaling by the IL-15. As N72D mutant IL-15 has reportedly shown a higher affinity to its receptor β (23), we introduced N72D mutation in the huIL-15 gene to achieve the higher affinity. Similarly, we introduced K322A mutation into the IgHg2 gene to delay the complement-mediated clearance for enhancing the residence/retention of chimeric IL-15. Both the above-mentioned and deployed mutations were confirmed by the Sanger sequencing (**Figures 2E, F**).

The contribution of IL-15 signal peptide (SP) in the down-regulation of its expression and poor expression in mammalian cells is well established (24, 25). Therefore, we replaced the IL-15 SP with IL-2 SP (24) to improve the production of chimeric IL-15, and for that pcDNA3.1 vector was modified and the IL-2 signal peptide was inserted immediately after CMV promoter. Mutant IL-15 CDS (342 bp; without IL-15 signal peptide) and mutant IgHg2 gene (993 bp) were sequentially inserted into the modified pcDNA after IL-2 SP for a final insert to be expressed as IL-2SP-IL-15CDS-IgHg2 chimeric protein (**Figure 2G**). The resultant plasmid was confirmed by digesting it with different combinations of restriction enzymes as shown in **Figure 2H**, 342 bp: IL-15, 981 bp: IgHg2, and 1,343: fusion genes, size: plasmid with IL-2 SP. These fragments were amplified by PCR and sequenced to confirm the sequences. FASTA sequences obtained from Sanger sequencing were compared and found 100% similar through the NCBI blast and SIM alignment.

### Stable Cell Line Generation, Purification, and Characterization of Chimeric IL-15

**Figure 3:**
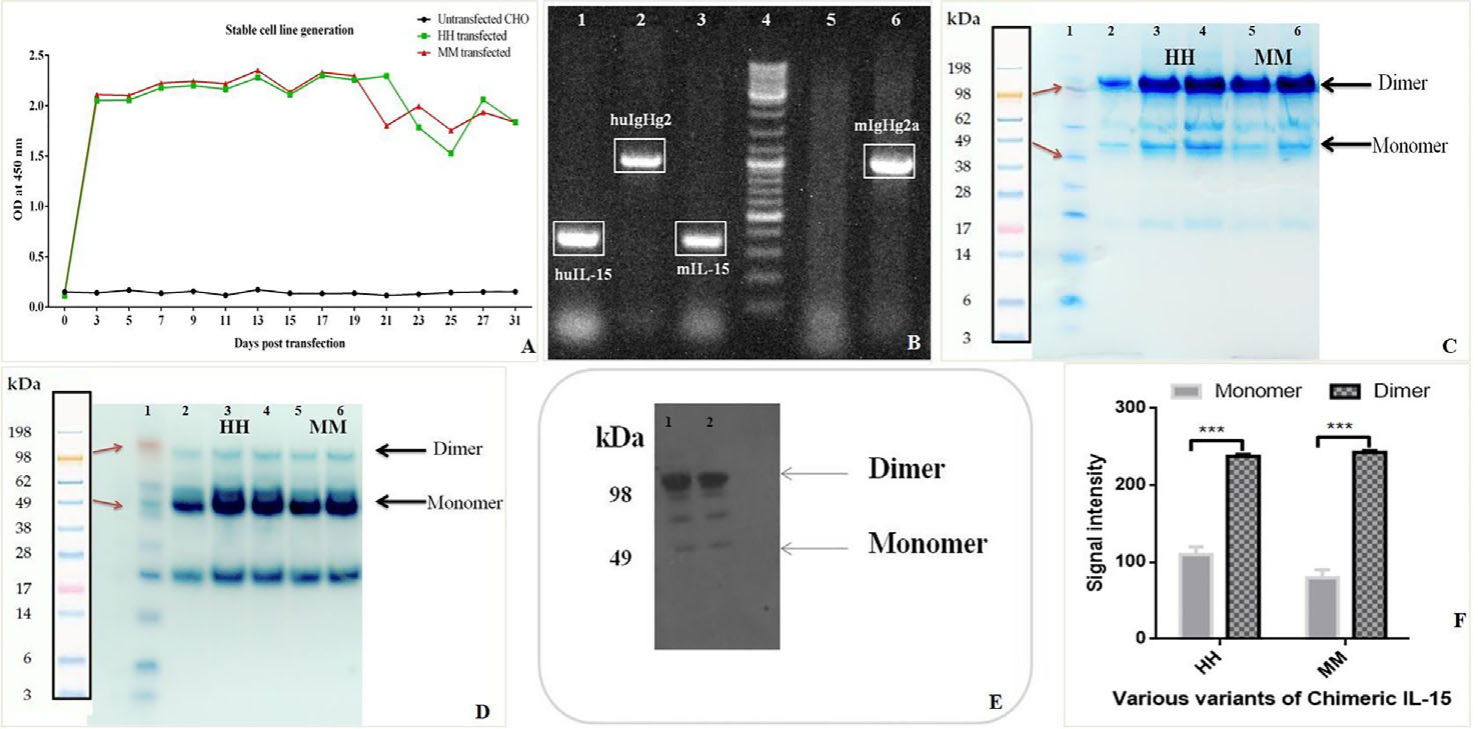
Production and Purification of human (HH) and mouse (MM)chimeric IL-15. **(A)** Sandwich ELISA of the supernatant from the chimera transfected cell lines was performed at various time points to confirm the stable cell line generation. **(B)** PCR amplifications of transfected genes from CHO cells were carried out at various time points by using IL-15 and IgHG2/2a specific primers to confirm the integration of genes (Well 4: Ladder, 5: Control (Untransfected CHO). **(C, D)** The proteins (HH: huIL-15 + huIgHG2 chimera, MM: mIL-15 + mIgHG2a chimera) were purified by using Agarose G column and loaded on SDS-PAGE under non-reducing **(C)** and under reducing conditions **(D)** suggesting the formation of dimer (Lane 1: Ladder, 3 & 4: HH chimera, 5 & 6: MM chimera). **(E)** Western blot analysis of HH and MM chimera (Lanes 1 & 2: HH chimera). **(F)** Densitometric analysis suggests the predominant presence of dimeric forms of chimeric IL-15(HH and MM). ***P ≤ 0.001.

Mammalian cell line (CHO) were transfected with Chimeric IL-15 construct or pcDNA3.1 vector by using Lipofectamine LTX plus reagents, with pEGFP-n1 plasmid transfection as a positive control. Green fluorescence was observed in CHO cells transfected with pEGFP-n1 plasmid 48 h post-transfection indicating the success of transfection. Nearly two-fold increased expression of chimeric IL-15 having IL-2SP was observed as compared to the chimeric IL-15 with IL-15SP. On day 3 post-transfection, CHO cells were fed with selective media containing geneticin antibiotic and geneticin kill curve for CHO cells were drawn; 400 µg/ml geneticin was the lowest concentration to kill all un-transfected cells within 7 days. ELISA results confirm the successful production of chimeric IL-15 (**Figure 3A**). The integration of transfected genes in the genome of CHO cells was detected by PCR amplification of the transfected genes (IL-15 and IgG2) (**Figure 3B**). CHO cells expressing chimeric IL-15 were successfully maintained in selection media for 60 days, and chimeric IL-15 was purified from cell-free supernatant by protein G affinity chromatography. Purified IL-15-IgHg2/2a preparation showed two bands with molecular weights ~98 kDa (prominent bands) and ~49 kDa with non-reducing SDS-PAGE (**Figure 3C**), wherea**s** purified IL-15-IgHg2/2a preparation showed one band of molecular weight ~49 kDa (**Figure 3D**) with reducing SDS-PAGE. The molecular weight determined by SDS-PAGE of homodimeric chimeric IL-15 was greater than the molecular weight calculated (98 kDa) based on the amino acid sequence of IL-15 and IgHg2/2a fusion proteins. The increased molecular weight could be due to glycosylation of the proteins expressed by CHO cells. The production of chimeric IL-15 was confirmed by western blot analysis (**Figure 3E**) HH or MM, and densitometric data also suggests that chimeric IL-15 forms the dimeric structure (**Figure 3F**).

### Coupling IL-15 to IgHg2/2a Base Increases the Half-Life and Serum Levels of IL-15

**Figure 4:**
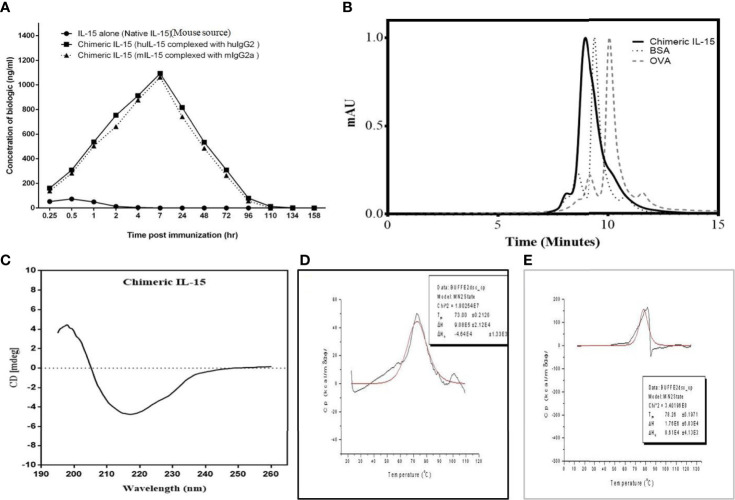
Characterization of chimeric IL-15. **(A)** Complexing IL-15 with IgHG2/2a increases half-life and bioavailability of exogenous IL-15 in the serum. Mice were injected with various biologics and were bled over time (0.25, 0.5, 1, 2, 4, 7, 24, 48, 72, 96, 110, 134 and 158 h after treatment), and presence of biologics was monitored using ELISA. **(B)** SE-HPLC chromatogram of HH suggests the predominant presence of dimeric structure. **(C)** Circular dichroism spectrum reveals that the HH chimeric IL-15 has properly folded structure. **(D, E)** Differential scanning calorimetry thermograms of MM **(D)** and HH **(E)** chimeric IL-15 depicts the Tmax = 78.26 and 73.00°C, analyzed by loading proteins on PerkinElmer Pyris1 DSC, suggest the chimeric IL-15 is highly stable in nature.

C57BL/6 mice were injected intraperitoneally with 2.5 μg/mouse native recombinant IL-15 or chimeric IL-15 reconstituted in PBS. Sera were collected at different time points before and after IL-15 administration. Our results show that the half-life of native IL-15 is ~1 h whereas the half-life of engineered chimeric IL-15 is ~40 h. With regard to maximum serum levels obtained, native IL-15 peaked at 30 min post-administration with a concentration of 73.5 ng/ml whereas chimeric IL-15 peaked at 7 h post-administration with a concentration of 1,050–1,100 ng/ml (**Figure 4A**). Furthermore, correlation of the availability of chimeric IL-15 with its functional activity was carried out by incubating splenocytes isolated from naive mice with serum collected at different time points. The increased expression of Bcl-2, STAT-3 and 5 was seen by q-PCR analysis (data not shown).

### Chimeric IL-15 Predominantly Forms the Dimeric Structure

Size exclusion-HPLC was used to resolve a dimer of chimeric IL-15 from the monomer. HPLC column was calibrated using standard proteins (OVA and BSA) of known molecular weight. Results obtained with HPLC chromatogram clearly indicate the presence of dimeric forms (**Figure 4B**).

### Chimeric IL-15 Comprises Properly Folded Structure

The secondary structure and protein folding of purified IL-15 was determined by circular dichroism (CD). Our data is suggestive of different structural elements showing characteristic CD spectra (**Figure 4C**), and negative bands at 222 and 208 nm indicate the presence of α-helical structure. Similarly, negative bands at 218 nm and positive bands at 195 nm attest to the well-defined antiparallel β-pleated sheets (β-helices) of chimeric IL-15. Also, we did not observe any negative bands near 195 nm which confirms the absence of disordered proteins on CD spectra of chimeric IL-15 (**Figure 4C**).

### Confirmation of Thermostability of Chimeric IL-15

The thermal and structural stability of chimeric IL-15 was confirmed by the differential scanning calorimetry (DSC). Thermal transition midpoint (Tm) or denaturation temperature (Tmax) values were generated by running various variants on PerkinElmer Pyris1 differential scanning calorimeter. Our data with higher Tm, as others (26–28), confirmed the increased conformational stability of chimeric IL-15. As the denaturation causes protein unfolding and might lead to the loss of therapeutic activity (29), achieving higher denaturation temperature (Tmax) has been a strategy associated with the therapeutic potential of pharmaceutical drug formulations. Tmax value for chimeric IL-15 was 73 and 78.26°C for the variants MM (mIL-15 and mIgG2a) and HH (huIL-15 and huIgG2) respectively (**Figures 4D, E**). Again, greater Tm of both variants attests to the stability of the protein.

### Structural Integrity and Functionality of Chimeric IL-15

**Figure 5:**
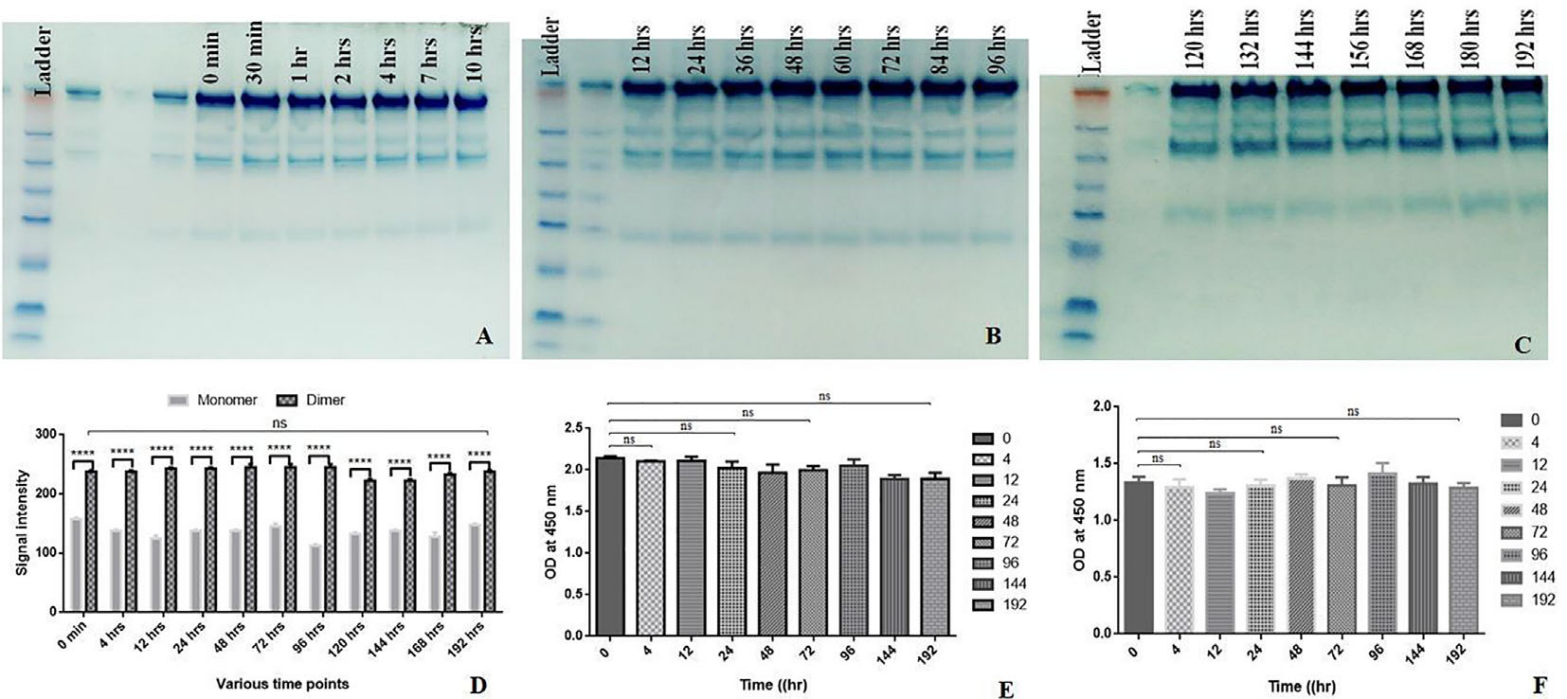
Shelf life assessment. HH Chimeric IL-15 was aliquoted in several 0.2 ml tubes and kept at 37°C. The tubes were taken out at various time points and stored in −20°C. **(A–C)** The SDS-PAGE analysis of chimera harvested at different time points. **(D)** Densitometry analysisof chimera harvested at different time points. **(E)** Anti-IL-15 and anti-IgG2 HRPantibody based sandwich ELISA of chimera harvested at different time points. **(F)** IL-15 Rβ based sandwich ELISAof chimera harvested at different time points. (Statistical analysis was performed in GraphPad Prism 6 by Tukey’s multiple comparisons test, 2-way ANOVA. ****P ≤ 0.0001, ns, not significant.

An effective biologic retains its functions upon storage over 7–8 days. Currently, there has been a shift in the approach of producing directly injectable therapeutic proteins from lyophilized form to liquid formulation. Therefore, it is imperative to produce structurally and functionally stable therapeutics upon being stored over months and maintaining a longer shelf-life (30). Aliquots of 0.2 ml chimeric IL-15 were kept at 37°C, followed by their storage at −20°C. The stability was confirmed by SDS-PAGE that is confirmatory of the structural integrity of chimeric IL-15 up to the experimental period of 192 h (**Figures 5A–D**). Moreover, sandwich ELISA results also confirmed the structural integrity/intactness of chimeric IL-15 as no significant changes were found in the absorbance at 0 min and at 192 h (**Figures 5E, F**). ELISA plates were coated with anti-IL-15 to capture antibody. As we know only if the IL-15 portion of chimeric IL-15 has the intact structure, it would be captured by anti-IL-15 antibody. Similarly, the IgG2 portion was detected by anti-IgG2 HRP antibody (**Figure 5E**). Based on our data, we confirmed the structural integrity of chimeric IL-15. Next, we wanted to check whether it retains its functionality with regard to the interaction with its receptor present on T cells. This was confirmed with the help of sandwich ELISA wherein the plates were coated with IL-15 Rβ, and hardly any significant changes were observed in absorbance (0 min to 192 h), suggesting that chimeric IL-15 was able to bind with its receptor and retained its functionality (**Figure 5F**).

### Immune-Modulation Activity of Chimeric IL-15

**Figure 6:**
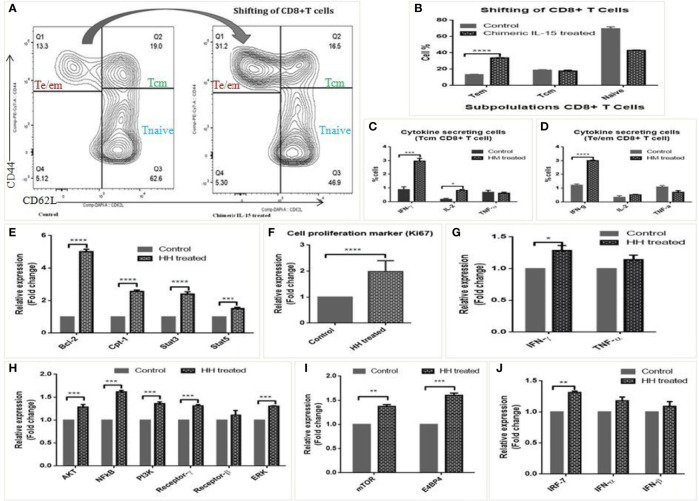
Biological activity of chimeric IL-15. **(A, B)** Representative flow-cytometry analysed layout of splenocytes untreated and treated with chimeric IL-15 (MM) **(A)**, and their percentage **(B)** suggests the shifting/modulation of T cells upon treatment. **(C, D)** Frequency of Te/em, Tcm and naive cells in control and chimeric IL-15 (MM) treated wells. **(E–J)** qPCR analysis suggests that chimeric IL-15 (HH) is able to activate IL-15 pathway and upregulates the expression of various genes involved in functionality of immune cells particularly T cells. The expression of IL-15 pathway genes (Bcl-2, Stat3, Stat5, NF-kB, IL-15 Rβ, Rγ), Cpt-1a (gene required for effector to memory T cell conversion), cell proliferation marker (Ki-67), T cell associated genes (IFN-γ, TNF-α, PI3K, ERK), NK cell activity genes (mTOR, E4BP4) and innate genes IRF-7, IFN-α, -β) were quantified by qPCR. (Statistical analysis was performed in GraphPad Prism 6 by Tukey’s multiple comparisons test, 2-way ANOVA. *p* < 0.05 is considered statistically significant). *P ≤ 0.05, **P ≤ 0.01, ***P ≤ 0.001, ****P ≤ 0.0001, ns, not significant.

The biological activity of chimeric IL-15 was tested through various *in vitro* and *in vivo* experiments. Splenocytes from OVA immunized mice were incubated with culture supernatants of chimeric IL-15 (MM) transfected CHO cells to assess the modulation of T cell response by flow cytometry. We found a significant (from 13.3 to 31.2%) increase in the number of Tem cells (**Figures 6A, B****)**, and greater frequency of IFN-γ (2 fold) & IL-2 (1.5 fold) producing cells (**Figures 6C, D****)**.

The biological activity was determined further with human PBMCs stimulated (12 or 24 h) with chimeric IL-15 (HH). qPCR analyses of various genes show that chimeric IL-15 modulates IL-15 mediated pathway. The expressions of Bcl-2, Cpt-1 and IL-15 mediated gene STAT3 were seen enhanced by 4, 1.5, and 1.5 fold, respectively (**Figure 6E**). Further, 2 fold increase in the expression of Ki-67 mRNA levels suggests the chimeric IL-15 induced cell proliferation (**Figure 6F**). Moreover, chimeric IL-15 mediated augmentation of T cell responses was confirmed by the significant upregulation seen in the transcripts levels of NF-kB, Akt, PI3k, ERK, IFN-γ, and TNF-α. Moreover, the higher expression of IL-15 Rγ and IL-15 Rβ was also seen (**Figures 6G, H****)**. As transcription factors, mTOR and E4BP4 are critical for survival/function of T cells (31), we saw chimeric IL-15 treated PBMCs were able to activate mTOR and E4BP4 (**Figure 6I**). Also, IL-15 is known to activate the IRF-7, a key controller of innate responses. Therefore, we looked for its expression and found that chimeric IL-15 activates the IRF-7 expression and its associated cytokines IFN-α, IFN-β as well (**Figure 6J**).

## Discussion

Interleukin (IL)-15 is a master regulator of survival, development, homeostasis, and activation of T, NK, & NK-T cells. This is required for various functions of B cells, DCs, macrophages, and mast cells (3) as well. IL-15 helps maintain naive, effector, and memory T cells, and plays an instrumental role in the generation and reactivation of antigen-experienced T cells (3, 32). This is of note that the presence of IL-15 provides signals for the long-term maintenance of Ag-specific memory CD8^+^ T cells in the absence of Ag challenge (33). The potential of IL-15 to reactivate the memory CD8^+^ T cells during the infectious challenge proves its adjuvanticity in vaccine development and cancer immunotherapy. IL-15 is shown to regress tumors by activating the tolerogenic tumor-infiltrating cells (TIL) (34, 35). IL-15 reportedly improves *in vivo* anti-tumor activity of adoptively transferred CD8^+^ T cells and expands self-reactive CD8^+^ T cells (36, 37). The exploration of potential adjuvanticity of IL-15 to combat various diseases including HIV, tuberculosis, and malaria (3, 5, 38, 39) is underway. Therefore, it has drawn significant attention to explore its therapeutic potential. However, its short half-life (40, 41) and poor bioavailability limit its therapeutic use. Further, the need of higher quantity for dosing and frequent dosing schedule to achieve the optimum therapeutic response (23) mounts upon the risks of adverse effects and cost-effectivity (41). The present study is an orchestrated attempt to increase stability, improve the half-life of IL-15 by engineering the chimeric IL-15 aiming at reducing the cost of treatment, and minimize side effects due to its inflammatory responses.

To overcome the above-mentioned limitations, we selected IgG2/2a as the base for the IL-15 chimera. IgG2 and IgG1 have a higher circulatory half-life (21 days), and IgG2 shows moderate to strong binding affinity with the Fc receptor. This activity is restricted largely to APC and not NK cells. IgG2 presents IL-15 to CD8^+^ T cell through APC and its effect is reflected in robust Ag-specific memory T cell response, which is required for vaccine development and anti-tumor therapy. The trans-presentation of improved chimeric IL-15 would be predominantly restricted to T cells by specifically binding to APC, and it might not trigger the inflammatory responses. IgG2 is poor in activating the complement system and the introduction of mutation K322A would further minimize the complement activation and delay the immune clearance, which in turn increases the residence time of chimeric IL-15.

IL-15 complex (15) is made through non-covalent interaction of IgG1-IL-15Rα and IL-15, but our chimeric IL-15 is covalently fused with the Ig base deploying recombinant DNA technology. IL-15 complex is very sensitive to the conditions of storage and transport, and would easily fall apart (unstable) creating a mixture of proteins. Our chimeric IL-15 however is a single stable molecule with less sensitivity to the storage condition. In fact, the results obtained from the DSC analysis supports our idea (**Figures 4D, E****)**.

Homodimeric monoclonal antibodies are shown more effective than their monomeric counterparts (19–21). Additionally, dimers are more stable and resistant to proteolytic cleavage than monomers. Also, low dose and lesser dosing frequency are required to maximize the therapeutic response. Therefore, we designed and predicted *in silico* constructs of chimeric IL-15 to achieve the dimerization. We confirmed the homodimer formation through *in silico* as well as wet-lab experiments. Our data showed that our chimeric IL-15 has the natural tendency to form dimers, and this property of chimeric IL-15 made it highly stable and we expect the biologic to be very efficacious.

Considering our objective of improving the efficacy of IL-15, we increased the affinity of IL-15 with its receptor on T cells in addition to its stability. Therefore, we introduced N72D mutation into IL-15 which is known to have a higher affinity for IL-15Rβ present on T cells (23). This insertion of mutation would facilitate quick and target specific presentation of IL-15.

Pharmacokinetic studies showed the enhanced (>40) half-life (residence time) of engineered chimeric IL-15. This is of particular importance that a reasonable level of chimeric IL-15 was detected in the serum 15-minute post-injection. This is suggestive of the rapid and longer availability of biological molecules for action, and chimeric IL-15 was seen 1,050–1,100 ng/ml at 7 h post-administration. Besides, *in vivo* half-life, thermal stability, and shelf-life of any biologics are equally important for conferring its therapeutic and immunogenic effect. Differential scanning calorimetric analysis advocates for the stability of chimeric IL-15 due to its high Tmax value. Moreover, chimeric IL-15 retained its structural integrity and functionality for a longer time at 37°C.

IL-15 activates JAK1 and JAK3 that phosphorylate STAT3 and STAT5, respectively. It results in phosphorylation of Src related tyrosine kinases and activation of anti-apoptotic protein Bcl-2 (3). While evaluating the biological activity of chimeric IL-15, we observed a significant increase in the levels of STAT3, STAT5, and Bcl-2 in human PBMCs. This suggests the activation of a typical IL-15 signaling cascade by the chimeric IL-15. IL-15 reportedly promotes the expression of Cpt1a, a mitochondrial enzyme required for the conversion of effector T cells into memory T cells (42) and maintenance of long-lasting T cell responses against pathogens by supporting the survival of CD8^+^ T cells (43). Our results corroborate the standing conclusion of the significant increase in transcripts levels of Cpt1a upon incubation of human PBMCs with chimeric IL-15 for 12/24 h. Further, we saw significant induction of IFN-α, -β, -γ, TNF- α; NF-κβ, AKT, PI3K, ERK, Receptor-β, -γ, mTOR, E4BP4, mcl-1 and SyK-1 to confirm the immunomodulatory role of the engineered chimeric IL-15.

The generation of a higher frequency of memory T cells is required for any effective vaccine. Strong CD8^+^ T cell response is a critical requirement to combat many diseases such as HIV, TB and Malaria. Moreover, the reactivation of tumor-infiltrating CD8^+^ T cells is crucially essential for tumor regression. IL-15 is known to play a central role in mediating the above responses (3). The effect of IL-15 on T cell modulation in the splenocytes treated with chimeric IL-15 was assessed. We saw CD8^+^ T cell response is modulated to play an instrumental role in the conversion of higher frequencies of IFN-γ and IL-2 expressing cells (**Figures 6C, D****)**. Similar results were achieved upon administering the pChimeric IL-15 with pOVA, which augment the magnitude of Ag-specific effector CD8^+^ T cells and generation of long-lived memory T cells. Our preliminary results suggest the safety of chimeric IL-15.

In essence, our results show the stable, long-lasting, and efficiently bioavailable engineered chimeric IL-15. The chimeric IL-15 is biologically active possessing immunomodulatory activity. Thus, our findings suggest that chimeric IL-15 can be used to induce CD8^+^ T cell proliferation, their function, and longevity. Also, our chimeric IL-15 might be a good adjuvant for developing the vaccine for targeting intracellular pathogens and cancers.

## Experimental Procedures

### *In Silico* Evaluation of Chimeric IL-15

The amino acid sequences of human as well as mouse IL-15 and IgG2 domains (CH1, Hinge, CH2, and CH3) were obtained from the Uniprot protein database (44). The monomeric and dimeric structures for IL-15-IgG2 (chimeric IL-15) were generated by I-TASSER (45) and COTH server (46) respectively. Wherever required, the predicted structures were improved by various ways e.g., reconstruction of protein backbone (47, 48), the addition of Hydrogens (49), fixation of side chains (50), and removal of steric clashes (51, 52). Moreover, several approaches e.g., kobamin (53), Modrefiner (54), and MDWeb (Amber, NAMD & Gromcas) (50) were adopted for energy minimization and molecular dynamics to obtain better modelled structures. Ramachandran plots were generated by Rampage (55) and Molprobity (49). Additionally, the QMEAN-, TM- and RMSD values were calculated by QMEAN Server (56–58), TM-score (59, 60), and Superpose server (61), respectively. ExPASy tool ProtParam (62) was used for physico-chemical analyses including *in vivo* half-life, instability index (an estimate of the stability of proteins), aliphatic index, and Grand average of hydropathicity (GRAVY) by providing the sequences in fusion forms. Chiron server (51, 52) was used for estimating the steric clashes, Hydrogen bonds (unsatisfied), Solvent accessible surface area, and Void volume. Molprobity server (49, 63) was used for calculating the Clash score, Poor rotamers, MolProbity score, and Cβ deviations. The docking of chimeric IL-15 with IL-15 receptor β was carried out by using the ZDOCK server (64) and Chimera tools (65). The intermolecular hydrogen bonds (the bonds between IL-15 portion of chimera and receptor β) were calculated by FindHBonds command of Chimera tool (65) and Intermolecular H-bonds command of Accelrys Discovery Studio Visualizer (66). Also, salt bridges were assessed by Find Salt Bridges options of Accelrys Discovery Studio Visualizer and looked for protein–protein interaction by AutoDock tools (67).

### Binding Affinity Prediction

Various docked structures (protein-protein complexes) were submitted to the PRODIGY (PROtein binding enerGY prediction) web server (68, 69) for the prediction of binding affinity and identification of biological interfaces from the docked structures. We submitted the IL-15–IL-15 Tri-receptor complex structure to the said server and predicted the binding free energy (Gibbs free energy, ΔG) and the dissociation constant (Kd) for IL-15-IL-15 Rα chain, IL-15–IL-15 Rβ chain, and IL-15–IL-15 Rγ chain.

### Animal Study

Female BALB/c and C57BL/6 mice were procured from Zydus Pharmaceuticals, Ahmedabad. These animals were housed in an Animal house Facility at the Institute of Pharmacy, Nirma University. Feed (*ad libitum*) and water were available in the animal facility. All experiments were performed in accordance with a protocol approved by the Institutional Animal care and use committee of Nirma University.

### Ethics Approval and Consent to Participate

All the methods were carried out according to the protocol approved by the Institutional Ethical Committee, Nirma University, Ahmedabad. Venous blood of healthy volunteers was collected following informed consent as per the ICMR guidelines, and PBMCs were used from heparinized blood for isolating RNA and amplifying the cDNA for cytokines.

### Amplification of IL-15 and IgG2 Constant Heavy Chain (IgHg2) From Human and Mouse Source

#### Amplification of huIL-15 and huIgHg2 Genes

Blood from healthy volunteers was collected in the EDTA vial. RNA was extracted from 0.5 ml fresh blood by RNA isolation kit (Thermo Fisher Scientific). The expression of huGAPDH, huIL-15, and huIgHg2 was determined by RT-PCR. Full-length huIL-15 (505 bp; CDS with signal peptide; without stop codon) and huIgHg2 (981 bp; CH1-Hinge-CH2-CH3; with stop codon) were amplified by using specific primers consisting of restriction enzyme recognition sequences (**Table 2**).

**Table 2:** Primer sequences used for fusion constructions in this study.

Primer	Order	Sequence (5’→3’)	Gene BankAccession No.
huIL-15	Forward	CACCGGTACCAACTGGGTGAATGTAATAAGTGATTTG	NM_172175
	Reverse	CGGGATCCAGAAGTGTTGATGAACATTTGGAC	
huIgHg2	Forward	CACCGGATCCGCCTCCACCAAGGGCCCAT	AJ250170
	Reverse	GCTCTAGATCATTTACCCGGAGACAGGGAGAGGCTCTTCTGT	
mIL-15	Forward	TAGGTACCGGCATTCATGTCTTCATTTTGG	NM_008357
	Reverse	TAGGATCCGGACGTGTTGATGAACATTTGG	
mIgHg2a	Forward	AGGATCCGCCAAAACAACAG	KC295246.1
	Reverse	TCTAGAATCATTTACCCGGAGTCC	

The nucleotide sequence for each gene was procured from the Gene Bank (NCBI). The Accession numbers were given for each sequence. The primers having restriction enzyme recognition sequences were designed by using PrimerQuest Tool (Integrated DNA Technologies) and OligoPerfect Designer (Thermo Fisher Scientific). The HPLC purified primers (Sigma Aldrich) were used.

#### Amplification of mIL-15 Gene

For induction of mIL-15 mRNA, Poly I:C (100 µg/200 µl PBS/mouse) was injected intravenously to female BALB/c mouse. Four hours later, splenocytes were harvested to extract RNA by RNA-isolation kit (Thermo Fisher Scientific, USA). β-actin and mIL-15 expression were determined by RT-PCR. Full length mIL-15 (483 bp) (without stop codon) was amplified by using specific primers having restriction enzyme recognition sequences (**Table 2**).

#### Amplification of mIgHg2a Gene

RNA was extracted from 10-2.16 (ATCC^®^ TIB-93™, a kind gift from Prof. NilabhShastri, UC Berkley) B cell hybridoma by RNA isolation kit (Thermo Fisher Scientific, USA). mβ-actin and mIgHg2a expression were determined by RT-PCR. mIgHg2a portion (1,007 bp) was amplified by using specific primers having restriction enzyme recognition sequences (**Table 2**).

### Chimeric Construct for Protein Expression

The amplified genes (IL-15 and IgHg2a) were separately cloned into PCR2.1 TOPO Vector (Thermo Fisher Scientific). K322A mutations into IgG2 Fc and N72D mutation into IL-15 were introduced by site-directed mutagenesis (Agilent). IL-2 signal peptide (SP) was inserted in pcDNA3.1 vector downstream of CMV promoter by using GeneArt Service, Thermo Fisher Scientific, USA. Sequentially IL-15 CDS and IgHg2 genes were inserted in the IL-2-pcDNA3.1 vector to make IL-2SP-N72DIL-15CDS-K322AIgHg2-pcDNA3.1 construct (chimeric IL-15). All cloning, mutagenesis, and fusion constructs were confirmed by Sanger sequencing.

### Expression of IL-15-IgG2/2a Fusion Protein in CHO Cells, Stable Cell Line Generation, Purification, and Characterization of Chimeric IL-15

#### Production of Chimeric IL-15

CHO cells were transfected with pEGFP-n1 plasmid (positive control), Chimeric IL-15 construct, or pcDNA3.1 vector by using Lipofectamine LTX plus reagents (Thermo Fisher Scientific). Secretion of chimeric IL-15 was quantified by sandwich ELISA. Briefly, 96-well high binding plates (Corning) were coated with Anti-huIL-15, anti-mIL-15 capture antibodies (Thermo Fisher Scientific, USA), or recombinant IL-15 receptor β (R & D) and incubated overnight at 4°C. Plates were washed and then blocked using PBS/1% BSA/0.2% Tween 20 (200 µl/well) for 2 h at RT. After incubation blocking solution was discarded. Cell-free supernatant was added to the wells (100 µl/well) and plates were incubated for 2 h at RT. After washing with PBS/0.05% Tween 20, biotinylated anti-IL-15 Ab (Peprotech) or anti-IgG2 HRP labeled antibodies (Thermo Fisher Scientific; Abcam) were added to the wells (100 µl/well; 1:1,000 dilutions) and incubated for 1 h at RT. Avidin-HRP-ABTS (Peprotech) or TMB-H_2_O_2_ (Thermo Fisher Scientific, USA) was used as the substrate to develop the assay reaction was stopped by using 0.16M H_2_SO_4_. Plates were read at OD 405 or OD 450 nm.

#### Stable Cell Line Generation

pcDNA3.1 vector expresses the neo gene which encodes the amino-glycoside 3’-phosphotransferase enzyme. This enzyme makes the mammalian cells resistant to geneticin antibiotics. Therefore, geneticin (G418) antibiotic (Thermo Fisher Scientific) was used for screening the transfected colonies. Before proceeding with stable cell line generation it is mandatory to perform a kill curve assay to determine the minimum effective G418 concentration required to kill non-resistant (or untransfected) cells. 10^6^ CHO cells/well were treated with varying concentrations of G418 i.e., 100 to 800 µg/ml, and cell growth and numbers were checked regularly. The minimum concentration required to kill all untransfected cells within 7 days was noted and used for stable cell line generation. CHO cells were transfected separately with various chimeric IL-15 constructs. After 24 h post-transfection media was changed and checked for cell viability. At 72 h post-transfection media was removed, cells were washed with PBS, and selection media containing geneticin antibiotic was added to cells. The transfected cells showed resistance to geneticin and grew well in selection media. The secretion of chimeric IL-15 was routinely measured by ELISA, SDS-PAGE, and Western blotting. The integration of transfected genes into the CHO genome was determined by PCR amplification of transfected genes (i.e., IL-15 and IgG2). For this routinely RNA was isolated from transfected and untransfected CHO cells and cDNA was synthesized.

#### Purification of Chimeric IL-15

As this is Ig based therapeutic protein, affinity chromatography was used to purify/concentrate the protein (Merck Millipore). 200 µl Protein G resin (50% slurry) was added to the base of the exchange device. Storage buffer was removed by centrifuging the device at 1,000×*g* for 1 min. About 500 µl of 1× Bind/Wash Buffer was added and centrifuged at 1,000*g* for 1 min. About ~9 ml cell-free supernatant was added on beads, mixed with resin by pipetting, and incubated with gentle agitation for 1 h at RT. The device was centrifuged at 1,000×*g* for 1 min to remove the unbound proteins. About 1.5 ml Bind/Wash Buffer was added and centrifuged at 1,000×*g* for 1 min to remove the impurities. About 1 ml Elution Buffer was added and mixed with resin. The bound protein (i.e., chimeric IL-15) was eluted and collected in the fresh tube by spinning the device at 4,000×*g* for 10 min. The eluted protein was mixed with 75 µl neutralization buffer to adjust the pH.

#### Determination of Dimer Formation

The purified chimeric IL-15 was loaded on SDS-PAGE under non-reducing or reducing conditions to confirm dimer formation. The coomassie dye was used to stain the protein bands. Densitometry analysis was performed to compare the formation of dimer vs. monomer. The chimeric IL-15 was also confirmed by western blot analysis.

#### Western Blot Analysis

To confirm the presence of chimeric IL-15 proteins western blotting was performed by direct indirect methods. In the indirect method, Anti-IL-15 IgG1 antibody was used as primary antibody and anti IgG1-HRP labeled antibody was used as secondary antibody. The iBlot^®^ 2 Gel Transfer Device (Dry transfer, Thermo Fishier Scientific) was used to transfer the protein on nitrocellulose membrane from the gel. The membrane with transferred proteins was then processed for blot development either by using iBind Flex Western Device (as per manufacture’s recommendations) or by manual protocol. The membrane was blocked by 3% BSA for 2 h at RT and then incubated overnight with anti-IL-15 IgG1 antibody (primary antibody). Next day, the membrane was washed and incubated with anti-IgG1-HRP labeled secondary antibody for 2 h. The blots were developed by either using ECL kit or TMB-for western blot.

#### HPLC Analysis

Analytical size-exclusion HPLC was performed for purified chimeric IL-15 by using the Agilent 1260 Infinity instrument equipped with a UV detector to further confirm the presence of dimeric forms. About 20 µl of 1 μg/ml chimeric IL-15 diluted into PBS was injected into a Zorbax Bio Series GF-250 column with dimensions of 9.4 (i.d.) × 250 mm (Agilent Technologies). Phosphate-buffered saline was used as the mobile phase and a 1 ml/min flow rate was maintained. The programme was run for 45 min. OVA and BSA were used as standards. The chromatograms were analyzed by the open lab (Agilent) and presented in form of overlap peaks of chimeric IL-15, OVA and BSA.

#### Circular Dichroism Spectra

The CD spectra of chimeric IL-15 was taken at room temperature (25°C) with a Model J-720 JASCO spectrometer at IIT Gandhinagar. A rectangular quartz cell with a path length of 0.1 mm was used. The blank scan was run from 260 to 195 nm with 0.22 µm filtered double distilled water. The scanning speed and bandwidth were set on 50 nm/min and 2 nm, respectively. Three accumulations or repeats were taken.

### Pharmacokinetic Analysis

#### Determination of Half-Life of Chimeric IL-15

Mice were injected intraperitoneally with 2.5 µg/mouse huIL-15, mIL-15 (2.5 µg) or various variant of chimeric IL-15 and were bled at various time points (0.25, 0.5, 1, 2, 4, 7, 24, 48, 72, 96, 110, 134 and 158 h after treatment), and ELISA was performed to monitor the level of injected biologics in mouse serum as per the protocol discussed earlier. The serum half-life of various biologics was calculated using the medical calculator provided by Cornell University as per the given instructions (40, 70). The mean residence time was calculated by Phoenix WinNonLin and PKsolver softwares (71).

#### Stability Assessment of Engineered Chimeric IL-15

Measurements of thermal stability were carried out using Microcal-VP differential scanning calorimeter at MBU-IISC Bangalore, as per manufacturer’s recommendations. Approximately 6 ml of 1 mg/ml proteins samples were carefully injected into the sample cell and PBS was used as the reference which is injected into the reference cell. The run was conducted in the temperature range 25–125°C in a nitrogen stream (flow rate at 20 ml/min, heating rate 1°C/min). The Thermal transition midpoint (Tm) or denaturation temperature (Tmax) or Tpeak was determined for various variants of chimeric IL-15.

#### Shelf-Life of Chimeric IL-15

Chimeric IL-15 was aliquoted in several 0.2 ml tubes and kept at 37°C. The tubes were taken out at various time points i.e., 0, 0.5, 1, 2, 4, 7, 10, 12, 24, 36, 48, 60, 72, 84, 96, 108, 120, 132, 144, 156, 168, 180 and 192 h and stored at −20°C. These fractions were loaded on SDS-PAGE to test the structural integrity or the ability of chimeric IL-15 to retain its structure. Densitometry was performed to determine the dimer: monomer ratio. Sandwich ELISA was performed to test the intactness of both partner proteins and to test the ability of chimeric IL-15 to retain its functionality (binding affinity to its receptor). The ability of chimeric IL-15 to retains its efficacy was tested by *in vitro* assays. Splenocytes were incubated with the above fractions for 12 h (1 µg/fraction/well). After 12 h of treatment, RNA was isolated, and cDNA was synthesized. qPCR analysis was carried out to quantify transcripts of Bcl-2, STAT-3, -5A, and cpt-1a expression.

### Flow Cytometry for Cell Surface Staining

Spleen of C57BL/6 mice immunized with OVA was harvested and minced to prepare the single cell suspension. RBCs were lysed with 2 ml ACK (Ammonium-Chloride Potassium) RBS lysis solution. Cells were counted by Countess II Automated Cell Counter (Thermo Fisher Scientific) and 2 × 10^6^ cells were incubated with cell free supernatant of transiently transfected with chimeric IL-15 construct or untransfected and processed for cells surface staining. After incubation, the cells were pelleted down and then resuspended in left over liquid. Cells were incubated with normal mouse serum (C57BL/6) acting as Fc block for 10 min at 4°C. After that, cells were added with the fluorescent antibodies for surface markers and incubated at 4°C for 20 min. Antibodies against CD3 (BD, 145-2C11 clone), CD8 (BD, 53-6.7 clone), CD44 (BD, IM7 clone), CD62L (BD, mel-14 clone) were used. Cells were then washed twice with FACS buffer at 400*g* for 5 min at 4°C. Cells were finally resuspended in 1% para-formaldehyde (PFA) before acquisition in a flow cytometer. Stained cells were acquired in FACS canto-II machine of BD Bioscience at Supratech Micropath laboratory, Ahmedabad, Gujarat. Data analysis was done using FlowJovX software.

### Determination of the Biological Activity of Chimeric IL-15

PBMCs were isolated from the human blood using Histopaque 1077. Final densities of 10^6^ cells/ml in RPMI with 10% serum were dispensed into 6 well plates at equal concentrations and the same were incubated with supernatant of CHO transfected with chimeric constructs or various concentrations of purified chimeric IL-15 protein (0.5 μg, 1 μg, or 2 μg/ml). Cells were harvested and RNA was isolated after 12 h of incubation. cDNA was prepared and q-PCR was carried out for measuring the expression levels of various genes involved in the IL-15 pathway and T cell modulation. Similarly, we evaluated the activity of the chimeric protein in splenocytes by gene expression analysis. Also, cellular response and intracellular cytokines were measured by flow cytometry.

### Data Analysis

Analysis of flow cytometer data was done using FlowJo vX software. Statistical analysis was performed in GraphPad Prism 6 by Tukey’s multiple comparisons test, 2-way ANOVA. p <0.05 is considered statistically significant.

## Data Availability Statement

The datasets presented in this article are not readily available, in accordance of not conflicting the interest of patent owners. However, except the cloned products, the remaining data could be made available by requesting to corresponding author. Requests to access the datasets should be directed to Sarat K. Dalai, sarat.dalai@nirmauni.ac.in.

## Ethics Statement

The studies involving human participants were reviewed and approved by Institutional Ethical Committee, Nirma University, Ahmedabad, Guajart, India-382481. The patients/participants provided their written informed consent to participate in this study. The animal study was reviewed and approved by Institutional Animal care and use committee, Nirma University, Ahmedabad, Gujarat, India-382481.

## Author Contributions

MP performed the experiments and analyzed data. NY helped in performing the experiments. SKD supervised the study. MP, NY and SKD wrote the manuscript. All authors contributed to the article and approved the submitted version.

## Conflict of Interest

MP, NY and SD have ownership interest in patent filed for improved chimeric IL-15 [Indian patent number: E-2/2703/2017/MUM; App. Number: 201721010096 (published)].
